# Reactive morphology of dividing microglia following kainic acid administration

**DOI:** 10.3389/fnins.2022.972138

**Published:** 2022-09-29

**Authors:** Tabitha R. F. Green, Sean M. Murphy, Maria P. Moreno-Montano, Etienne Audinat, Rachel K. Rowe

**Affiliations:** ^1^Department of Child Health, University of Arizona College of Medicine - Phoenix, Phoenix, AZ, United States; ^2^Institute of Functional Genomics (IGF), University of Montpellier, CNRS, INSERM, Montpellier, France; ^3^Department of Integrative Physiology, University of Colorado, Boulder, CO, United States

**Keywords:** cell division, proliferation, microglial reactivity, inflammation, kainate (KA)

## Abstract

The microglial response to a pathological microenvironment is hallmarked by a change in cellular morphology. Following a pathological stimulus, microglia become reactive and simultaneously divide to create daughter cells. Although a wide array of microglial morphologies has been observed, the exact functions of these distinct morphologies are unknown, as are the morphology and reactivity status of dividing microglia. In this study, we used kainic acid to trigger microglial activation and cell division. Following a cortical kainic acid injection, microglial morphology and proliferation were examined at 3 days post-injection using immunohistochemistry for ionized calcium binding adapter molecule 1 (Iba1) to stain for microglia, and KI67 as a marker of cell division. Individual microglial cells were isolated from photomicrographs and skeletal and fractal analyses were used to examine cell size and spatial complexity. We examined the morphology of microglia in both wildtype and microglia-specific tumor necrosis factor (TNF)-α knockout mice. Data were analyzed using generalized linear mixed models or a two-way ANOVA. We found that dividing microglia had a more reactive morphology (larger cell body area, longer cell perimeter, and less ramification) compared to microglia that were not dividing, regardless of microglial release of TNF-α. However, we also observed dividing microglia with a complex, more ramified morphology. Changes in microglial morphology and division were greatest near the kainic acid injection site. This study uses robust and quantitative techniques to better understand microglial cell division, morphology, and population dynamics, which are essential for the development of novel therapeutics that target microglia.

## Graphical Abstract

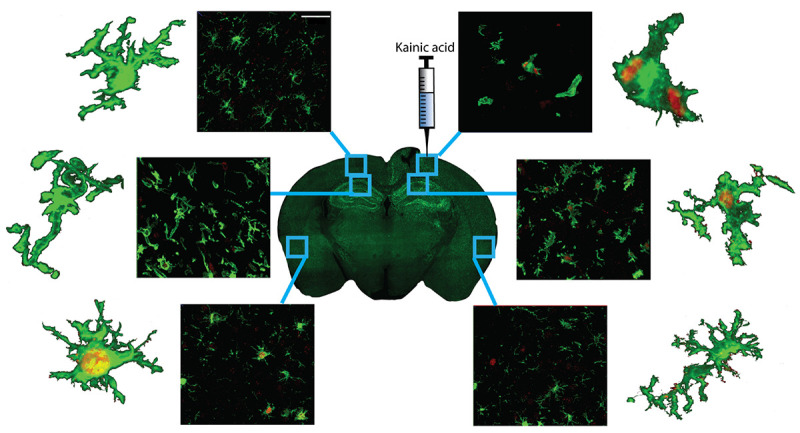

## Introduction

Microglia are critical immune cells localized to the central nervous system (CNS) (Kreutzberg, 1996). Through fast movement of their branches, microglia actively survey the CNS (Davalos et al., 2005; Nimmerjahn et al., 2005). This scanning activity allows microglia to rapidly detect abnormal perturbations in the microenvironment that are associated with many types of injury or infection (Morrison et al., 2017; George et al., 2019; Mbagwu et al., 2019; Green et al., 2021). Upon detecting neuropathological stimuli, microglia quickly respond and attempt to protect the brain from further injury (Wake et al., 2011; Eyo et al., 2021). The microglial response is initially beneficial in helping clear cellular destruction caused by an injury or infection. However, a chronic microglial response is often pathological and can cause additional damage to the brain *via* excessive release of pro-inflammatory intermediates (Badoer, 2010; Lull and Block, 2010; Loane and Kumar, 2016).

The microglial response to a pathological microenvironment is hallmarked by a change in cellular morphology and proliferation (Morrison et al., 2017; Savage et al., 2019). Reactive microglia retract their processes and adopt a less complex branching structure and an enlarged cell body (Doorn et al., 2014; Morrison et al., 2017; York et al., 2018; Savage et al., 2019; Green et al., 2021). In severe cases, microglia adopt an ameboid morphology, much like circulating macrophages (Doorn et al., 2014). Because of these distinctive morphological changes, microglial morphology is often used as a marker of inflammation and severity of tissue damage (Davis et al., 1994; Minlebaev et al., 2013; Morrison et al., 2017). Although a wide array of microglial morphologies has been documented, the exact functions of these distinct morphologies are unknown, as are the morphology and reactivity status of microglia undergoing cell division. To reduce some of the harmful effects caused by chronic microglial reactivity, the population dynamics and morphologies of reactive and dividing microglia must first be investigated using robust and quantitative techniques. Precise microglial morphological data will allow specific therapeutic targeting of pathology-associated microglia and provide an accurate measurable outcome for preclinical drug screening.

Tumor necrosis factor-α (TNF-α) is a key cytokine that microglia release in response to inflammatory stimuli (Kuno et al., 2005; Smith et al., 2012). TNF-α can act on microglia *via* an autocrine mechanism that increases activation and the subsequent release of pro-inflammatory cytokines, including additional TNF-α (Kuno et al., 2005). Initial release of TNF-α by microglia may be beneficial, as TNF-α supports neuronal function, upregulates inflammatory pathways, and promotes neuroprotection (Carlson et al., 1999; Stellwagen and Malenka, 2006; Morrison et al., 2017). However, the chronic release of TNF-α caused by acquired neurological injuries, neurodegenerative disease, and infections, exacerbates neuropathology and worsens outcomes (Badoer, 2010; Lull and Block, 2010; Smith et al., 2012; Loane and Kumar, 2016). This secondary damage can include a prolonged inflammatory response, increased permeability of the blood-brain barrier, cytotoxicity, apoptosis, excitotoxity, and demyelination (Kuno et al., 2005), all of which prolong microglial reactivity (Smith et al., 2012). TNF-α is also involved with signaling pathways that control cell division in a variety of cell types (Cao et al., 2021; Niu et al., 2021; Skartsis et al., 2021). We hypothesized that eliminating TNF-α from microglia by genetic knockout (KO) would reduce microglial cell division and activation following an inflammatory trigger.

In this study, we used kainic acid to trigger microglial reactivity and cell division (Chang et al., 2006; Christensen et al., 2006; Akahoshi et al., 2007; Avignone et al., 2008; Ulmann et al., 2013; Bosco et al., 2018; Morin-Brureau et al., 2018; Araki et al., 2020; Di Nunzio et al., 2021). We followed a procedure of unilateral intracortical kainic acid injection that was developed as a model of temporal lobe epilepsy with hippocampal sclerosis (Bedner et al., 2015). Following a cortical kainic acid injection, microglial morphology and division were examined using quantitative measurements of cell size and spatial complexity. Microglial cells were stained with anti-ionized binding adapter molecule 1 (Iba1), a specific marker of microglia, and dividing cells were stained with anti-KI67. KI67, a protein that is essential to form the perichromosomal layer during mitosis (Pyo et al., 2016; Hayashi et al., 2017), was used to label cells in the growth 2 (G2) and mitosis phases of the cell cycle (Uxa et al., 2021). Microglial morphology and division were quantified, in both TNF-α KO and wildtype (WT) mice, to investigate whether the genetic elimination of microglia-produced TNF-α altered microglial reactivity. We hypothesized that dividing microglia would be reactive, with a less ramified morphology, compared to non-dividing microglia after kainic acid induced inflammation. Furthermore, we hypothesized that there would be less microglial reactivity and division in TNF-α KO mice.

## Materials and methods

### Rigor

To ensure all experiments were carried out under blinded conditions, animal numbers were re-labeled by an investigator not associated with the experiments and were revealed after all data collection was completed. All experiments were approved by the ethics committee of Languedoc Roussillon n°36 and the French Ministry of Research (APAFIS#9899-2017042514488653 v4). All experiments followed the guidelines of the European Union for the care and use of laboratory animals (council directive 2010/63/EU). The Animal Research: Reporting of *in vivo* Experiments guidelines were followed in the preparation of this manuscript. Pre-determined exclusion criteria included exclusion of mice that lost > 20% of their body weight or had unmanageable pain; however, none of the mice in the study met these criteria and, therefore, none were excluded. A total of 268 microglia from five TNF-α KO mice, and 210 microglia from four WT mice were randomly selected from photomicrographs (using coordinates and a random number generator) and were used for analyses. Microglia were selected regardless of whether the cell was KI67^+^ or KI67^–^.

### Animals

Adult (90–120 days old) male and female WT and TNF-α KO mice (C57BL/6J) from our in-house colony were used for all experiments. Mice were housed in a 12-h light: 12-h dark cycle at constant temperature with food and water available *ad libitum*. CX3CR1-CreER mice (Yona et al., 2013) were crossed with TNF-α*^flox/flox^* mice (Grivennikov et al., 2005) to generate Cx3cr1*^creER/+^*:TNF-α^+/+^ and Cx3cr1*^creER/+^*:TNF-α*^flox/flox^* littermates. CreER mediated silencing of TNF-α was induced by intraperitoneal (IP) injection of tamoxifen (Sigma-Aldrich T5648) dissolved in corn oil, every 24 h for 3 days, at a dose of 100 mg/kg body weight (Jahn et al., 2018). Mice were used for experiments 3 weeks after the last tamoxifen injection. Following the tamoxifen injection and post-surgery, mice were monitored post-operatively for pain and changes in weight.

### Surgical preparation and kainic acid injection

Mice were anesthetized with Domitor (1.2 mg/kg) and ketamine (80 mg/kg) *via* IP injection. Mice were secured in a stereotaxic headframe and a midline incision was made to expose the skull. A 1-mm craniectomy was performed at 2 mm posterior to bregma and 1.5 mm from the sagittal suture. A 10 μl beveled tip microsyringe (NanoFil, NF33BV, 33GA) attached to a microsyringe pump controller (Micro4) was lowered and 70 nL of 20-mM kainic acid (1 nmol, Sigma-Aldrich; dissolved in 0.9% NaCl) was injected above the left dorsal hippocampus, 1.7 mm beneath the skull surface at a constant rate (70 nl per minute). The needle was left in place for an additional 3 min after injection to reduce backflow through the needle tract. Collectively, these methods ensured all animals received kainic acid in precisely the same injection site. Incisions were sutured and the mice recovered on a heating pad until ambulatory. The mice were visually monitored for seizure-like behavior for the full duration of the post-operative period. Seizure-like behaviors were observed post-injection in all kainic acid-treated mice.

### Perfusion

At 3 days post-injection, a lethal dose of Euthasol^®^ (340 mg/kg) was administered. Mice were transcardially perfused with phosphate buffered saline (PBS, 10 ml), followed by 4% paraformaldehyde (PFA; 10 ml). Brains were harvested from the skulls and drop fixed in 4% PFA for 24 h. Brains were then transferred to 0.01% sodium azide solution for the following 24 h. Brains were sectioned at 40 μm using a vibratome and collected in wells of 0.01% sodium azide, where they were stored until they were stained.

### Immunohistochemistry

#### Regions of interest

**FIGURE 1:**
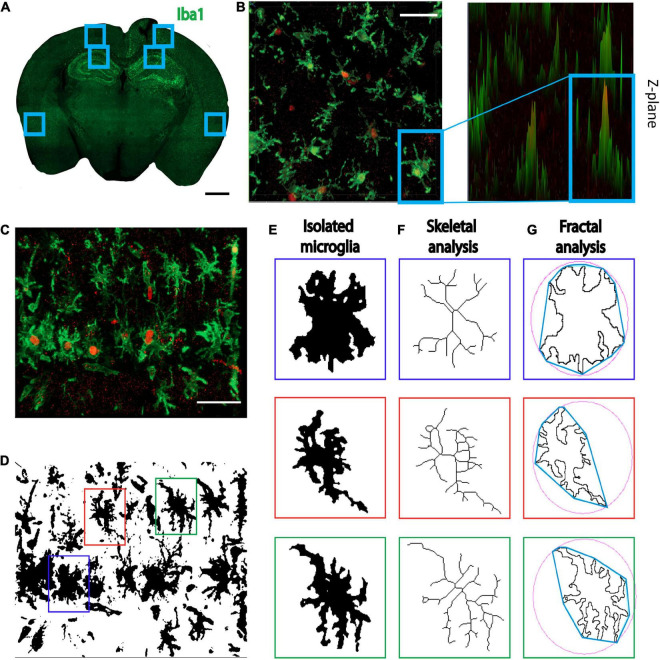
Methods for individual microglia ImageJ analyses. **(A)** Three regions of interest were selected, the cortex adjacent to the injection site, the CA1, and the perirhinal cortex. Scale bar = 1000 μm. **(B)** Iba1^+^/KI67^+^ co-labeled cells. Images were also displayed at 2.5 renderings to ensure all co-labeled cells were counted. Scale bar = 50 μm. **(C)** Representative image of 40× Z-stacked photomicrograph of Iba1 and KI67 staining used for single cell morphology analyses. Scale bar = 50 μm. **(D)** Photomicrographs were converted to binary and **(E)** 3 randomly selected microglia were isolated. **(F)** For skeletal analyses, cells were converted to skeletons and analyzed. **(G)** For fractal analyses, the isolated microglia were converted to outlines and analyzed. Red = KI67 staining; Green = Iba1 stain.

Three brain sections per mouse were randomly selected for each region of interest. The injection site was identified using a brightfield microscope (total area of 120 μm per brain was analyzed). Kainic acid injections are commonly used in epilepsy research to induce seizure-like activity in mice (Le Duigou et al., 2005, 2008; Avignone et al., 2008; Li and Liu, 2019). In this study, kainic acid was used to trigger microglia both locally and in response to seizure-like activity. Therefore, regions of interest included: (1) the cortex near the injection site, because of its high exposure to kainic acid; (2) the hippocampus (CA1), a key area involved in kainic acid-induced seizures which consequently becomes sclerotic (Bedner et al., 2015); and (3) the perirhinal cortex, as a distal region with a lower exposure to activating stimuli (Figure 1A).

#### Iba1 and KI67

Anti-Iba1 antibody was used to mark all microglia in the brain, regardless of their stage of the cell cycle (Morrison et al., 2017; Green et al., 2021). Anti-KI67 antibody was used to stain all cells undergoing cell division (Kuno et al., 2005; Inwald et al., 2013; Pyo et al., 2016). Cells that were co-labeled for Iba1 and KI67 were considered “dividing microglia” in this study. To maximize scientific rigor and allow accurate comparisons between samples, all staining was performed in a single round with the same aliquots of reagents. Tissue slices were selected and placed into 1 ml of 1X PBS in a 24 well plate. Slices were washed three times for 10 min on an agitator. Blocking solution [4% normal donkey serum (NDS, Sigma–Aldrich) and 0.2% Triton in 1X PBS] was applied for 2 h under agitation at room temperature. Primary antibodies [anti-Iba1 (WAKO rabbit Iba1, NC1801858) at 1:500 concentration and anti-KI67 (R&D systems AF7649) at 1:250, 2% NDS, and 0.2% triton in PBS] was applied to tissue and left to incubate overnight at 4°C. Tissue was then washed in PBS 3 times for 10 min, and incubated at room temperature with secondary antibody solution for 2 h [donkey anti-rabbit (Alexa 488 1:250, Invitrogen, A-110391:250), donkey anti-sheep (NorthernLights 557 1:250, R&D systems NL010), 2% NDS in 1X PBS]. Tissue was washed in PBS 3 times for 10 min under agitation and the tissue slices were mounted onto permafrost slides and coverslipped using DAKO fluorescent mounting medium.

#### Imaging

Z-stacked images (15 slices per image) in pre-determined regions of interest were taken using a 40× objective lens and a ZEISS ApoTome.2 with consistent exposure, numerical aperture, and apotome function (3 ApoTome grid shifts per image to increase axial resolution). Nyquist theorem was followed to ensure the signal adequately represented the biological samples. For full brain images, the ZEISS Axioscan Z1 was used to acquire Z-stack images (5 Z-stack planes) with a 20× objective. Microscope settings were pre-determined, and all tissue was imaged in one session under identical conditions. A total of 162 images were used for the 40× field of view analyses. We used 27 full brain slices for hippocampal cell counts (Supplementary Figure 1).

### Quantitative microglia morphology analyses

Zen Blue software was used to count the cells in the representative slices of the hippocampus from full brain images (Supplementary Figure 1). The 40× field of view cell counts were performed manually in ImageJ, and images were displayed using the Zen Blue 2.5-dimension function to ensure KI67 could be clearly visualized (Figure 1B). For all individual microglia morphological analyses, 40× Z-stacks (Figure 1C) were analyzed in ImageJ. For single cell analyses, images were binarized and three individual microglia were selected per image using image coordinates and a random number generator (Figure 1D). Using the region of interest tool, we isolated each selected cell from the binary image. We then used the paintbrush tool to remove any fragments that were not attached to the cell and connect any branches that had become fragmented because of image processing, using the original photomicrograph for reference. Isolated microglia were then converted to a skeleton (Figure 1E) and an outline (Figure 1F). The skeletal analysis plugin was run on individual skeletonized cells to measure the number of branches, branch length, and endpoints per microglia. Additionally, the ImageJ FracLac plugin, with added hull and circle results, was used to analyze the outlines of individual cells (Figure 1G; Morrison et al., 2017). The outcome measures of fractal analysis are: (1) density of pixels per individual cell; (2) fractal dimension (box-dimension; Db) defined as a statistical measure of the complexity of the microglial branches (Karperien et al., 2013); (3) circularity where a score of 1 represents a perfect circle; (4) lacunarity which is a geometric measure of the pattern complexity created by the branches and gaps to assess cell complexity (Karperien et al., 2013);and (5) span ratio used to measure cell shape and elongation which is calculated as the convex hull longest length divided by convex hull longest width (Morrison et al., 2017). The multipoint area selection tool in ImageJ was used to measure microglial cell body perimeter and area (Green et al., 2021).

### Statistical analyses

Prior to statistical analyses, Grubb’s outlier tests were run using the GraphPad outlier calculator and data points that were statistically significant (*p* < 0.05) outliers were excluded from further analyses. All measurements from isolated single cell microglia analyses had the following group sizes: WT KI67^–^ 140 microglia, TNF-α KO KI67^–^ 182 microglia, WT KI67^+^ 72 microglia, TNF-α KO KI67^+^ 88 microglia. For isolated single cell analyses, the microglia were randomly selected, independent of KI67 staining, resulting in differences in groups sizes between KI67^+^ and KI67^–^ groups.

Microglial cell count and cell body perimeter and area data from each region of interest (where an average value calculated from three photomicrographs per animal was considered) were analyzed using a two-way ANOVA followed by a Tukey’s multiple comparisons test when appropriate. Prior to ANOVAs, groups were assessed for equal variances to ensure the primary assumption of homoscedasticity was met. ANOVAs were conducted using GraphPad Prism 9.3.1.

For single cell data, we fit generalized linear mixed models to test for effects of KI67, genotype, and brain region (Stroup, 2012; Faraway, 2016). Because 54 data points were obtained for each mouse, we specified random intercepts for individual mice in all models to account for clustering and potential correlation among observations for a given mouse. For each outcome, we fit three mixed models: (1) a contralateral hemisphere-specific model that included a three-way interaction among KI67, genotype, and brain region; (2) an ipsilateral hemisphere-specific model that included a three-way interaction among KI67, genotype, and brain region; and (3) a non-hemisphere-specific and non-region-specific model (i.e., hemisphere and region data combined) that included a two-way interaction between KI67 and genotype. Collectively, these three models allowed us to investigate differences between hemispheres, among regions within hemispheres, between genotypes within and among regions within each hemisphere, and between KI67 positive and negative within and between genotypes within and among regions within each hemisphere. We did not fit a model with a four-way interaction among KI67, genotype, brain region, and hemisphere because such a model would have been overparameterized relative to the design effective sample size.

Exploratory analyses suggested that, depending on the outcome variable, different error distributions were needed in the mixed models to accurately reflect the data scales and to obtain reliable estimates. The fractal dimension outcome was approximately normally distributed, so we specified Gaussian error distributions. In contrast, span ratio was severely left-skewed, so we specified a Gamma error distribution with a log link function (i.e., multiplicative arithmetic mean model) (Faraway, 2016). Because lacunarity, density, and circularity were all proportions bounded between zero and one, we specified Beta error distributions (Ferrari and Cribari-Neto, 2004). Point voxels, number of branches, branch lengths, cell areas, and cell perimeters were all count variables that exhibited overdispersion (dispersion range: 5.78–51.47), so we specified negative-binomial error distributions (Hardin and Hilbe, 2018). We fit all models *via* the glmmTMB package in the R statistical computing environment (Brooks et al., 2017; R Core Team., 2022). We present results as the estimated marginal means with corresponding 95% confidence intervals (CI), as well as *p*-values for contrasts that were obtained following Tukey’s adjustments for multiple comparisons (Dunn, 1961), all of which we produced using the R package emmeans (Lenth, 2021).

Single cell multivariate data were presented as a heatmap using ClustVis (Metsalu and Vilo, 2015). All cells were included in the heatmap regardless of region or hemisphere and were separated by whether they expressed KI67. Five outcome measures that represent microglial reactivity were included in the heatmap: Endpoints per microglia, branches per microglia, total branch length per microglia, cell body area, and cell body perimeter. We observed no statistical differences in these outcome measures between genotypes, so we included all isolated microglia (KI67^–^ *n* = 320, KI67^+^ *n* = 158), regardless of genotype. All continuous data were standardized to have mean of zero and unit variance prior to plotting.

Prior to statistical analyses, a Type I error rate of α < 0.05 was pre-determined as an acceptable threshold. All data analyzed with two-way ANOVAs are presented as individual data points with the mean ± standard error of the mean (SEM). In contrast, all data analyzed with mixed models are displayed as individual data points with the point estimates and corresponding 95% confidence intervals.

## Results

### The number of dividing microglia was greatest near the injection site

**FIGURE 2:**
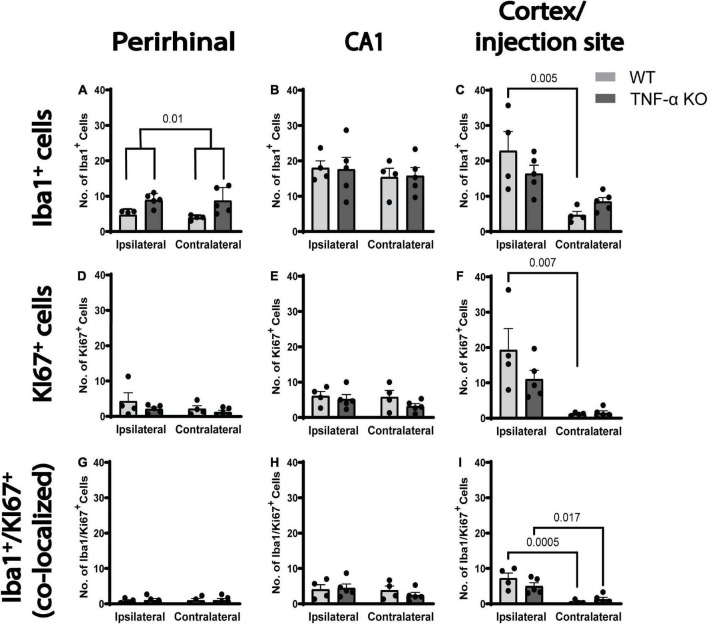
There were more dividing microglia per field of view in the ipsilateral cortex injection site in both WT and TNF-α KO mice. Cells were quantified in three images and averaged per animal for three regions of interest. **(A)** Average number of Iba1^+^ cells (microglia) in the perirhinal cortex, **(B)** CA1, and **(C)** cortex/injection site. **(D)** Average number of KI67^+^ cells (dividing cells) in the perirhinal cortex, **(E)** CA1, and **(F)** cortex/injection site. **(G)** Average number of co-labeled Iba1^+^ and KI67^+^ (dividing microglia) in the perirhinal cortex, **(H)** CA1, and **(I)** cortex/injection site. Data are presented as mean ± SEM. Data were analyzed using a two-way ANOVA (see Table 1). Statistically significant *post hoc* comparisons are indicated by bars with corresponding *p*-values. WT *n* = 4; TNF-α KO *n* = 5.

We quantified the number, the morphology and the proliferation of microglia in three different brain regions 3 days after a unilateral intracortical injection of kainic acid in mice. There were more Iba1^+^ microglia per field of view in the perirhinal cortex of TNF-α KO mice compared to WT mice (Figure 2A; see Table 1 for corresponding *p*-values and F-statistics). There were no differences in the total number of microglia in the perirhinal cortex (Figure 2A) or the CA1 (Figure 2B) between ipsilateral and contralateral hemispheres. However, there were more microglia in the ipsilateral cortex/injection site compared to the contralateral in WT mice (Figure 2C). There were no differences in the total number of Ki67^+^ dividing cells between hemisphere or genotype in the perirhinal cortex or CA1 (Figures 2D–E). There were more dividing cells in the ipsilateral cortex/injection site of WT mice than in the contralateral hemisphere (Figure 2F). KI67 labels all dividing cells and is not specific to dividing microglia. Therefore, KI67 cell counts represent any cell type that may divide after a kainic acid injection (e.g., astrocytes, oligodendrocytes, pericytes). There were no differences in the number of dividing microglia (Iba1^+^, KI67^+^) between hemisphere or genotype in the perirhinal cortex (Figure 2G) or the CA1 (Figure 2H). However, in the cortex/injection site, there were more dividing microglia on the ipsilateral side than the contralateral side (Figure 2I) in both WT and TNF-α KO mice. There were no differences in the number of microglia, total number of dividing cells, or number of dividing microglia between hemisphere or genotype when four 40 μm slices of the hippocampus were counted for Iba1^+^, KI67^+^, and Iba1^+^ and KI67^+^ cells (Supplementary Figures 1A–C).

**TABLE 1:** ANOVA results from cell count data (Figure 2) separated by brain region, histological stain, and effect.

Effect	Perirhinal			CA1			Cortex/injection site		
	Iba1	KI67	Iba1 × KI67	Iba1	KI67	Iba1 × KI67	Iba1	KI67	Iba1 × KI67
Hemisphere	*F*_1,14_ = 0.21 *p* = 0.65	*F*_1,14_ = 1.62 *p* = 0.22	*F*_1,14_ = 0.01 *p* = 0.92	*F*_1,14_ = 0.64 *p* = 0.44	*F*_1,14_ = 0.76 *p* = 0.40	*F*_1,14_ = 1.02 *p* = 0.33	*F*_1,14_ = 20.64 ***p* < 0.01**	*F*_1,14_ = 21.09 ***p* < 0.01**	*F*_1,14_ = 32.29 ***p* < 0.01**
Genotype	*F*_1,14_ = 16.17 ***p* < 0.01**	*F*_1,14_ = 1.70 *p* = 0.21	*F*_1,14_ = 0.06 *p* = 0.81	*F*_1,14_ < 0.01 *p* = 0.99	*F*_1,14_ = 1.87 *p* = 0.19	*F*_1,14_ = 0.20 *p* = 0.67	*F*_1,14_ = 0.21 *p* = 0.65	*F*_1,14_ = 1.74 *p* = 0.21	*F*_1,14_ = 0.65 *p* = 0.44
Interaction	*F*_1,14_ = 0.08 *p* = 0.78	*F*_1,14_ = 0.30 *p* = 0.59	*F*_1,14_ = 0.06 *p* = 0.81	*F*_1,14_ = 0.02 *p* = 0.89	*F*_1,14_ = 0.46 *p* = 0.51	*F*_1,14_ = 0.62 *p* = 0.45	*F*_1,14_ = 3.17 *p* = 0.10	*F*_1,14_ = 2.01 *p* = 0.18	*F*_1,14_ = 2.67 *p* = 0.12

Statistically significant results are in bold text.

### Dividing microglia had a larger cell body area and longer perimeter compared to microglia that were not dividing

**FIGURE 3:**
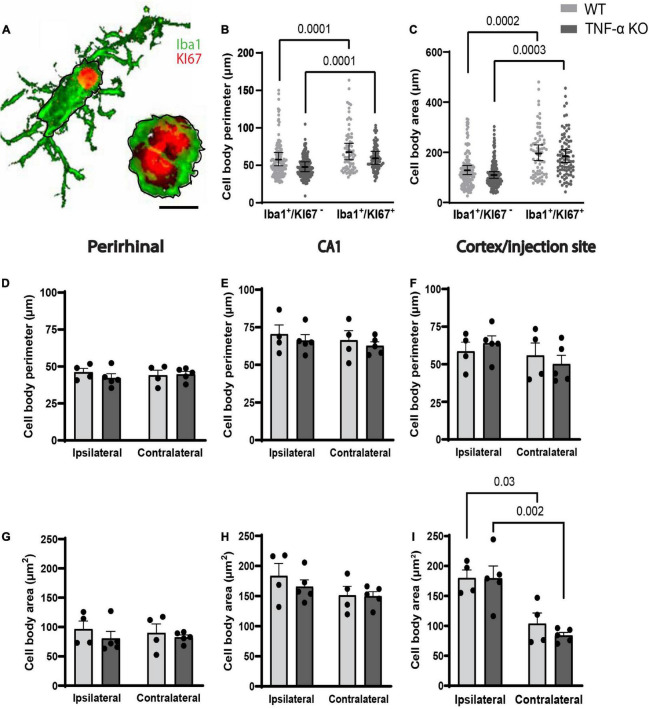
Dividing microglia had larger cell body areas and longer perimeters compared to microglia that were not dividing. **(A)** Iba1^+^ microglia were randomly selected, and the area and perimeter were traced and calculated in ImageJ (represented by black lines around the cell bodies). Scale bar = approximately 10 μm. **(B)** Cell body perimeter and **(C)** cell body area of individual microglia. **(B,C)** Individual data points are presented as dots and were analyzed using linear mixed models. Results are presented as point estimates with 95% confidence intervals (see Table 2). Statistically significant *post hoc* comparisons are indicated by bars with corresponding *p*-values. Iba1^+^/KI67^+^ = colocalization of Iba1 and KI67. TNF-α KO KI67^–^ *n* = 181 microglia; WT KI67^–^ *n* = 139 microglia; TNF-α KO KI67^+^ *n* = 87 microglia; WT KI67^+^ *n* = 71 microglia. **(D–I)** Microglia were quantified in three images and averaged per animal for three regions of interest. **(D)** Average cell body perimeter of microglia in the perirhinal cortex, **(E)** CA1, and **(F)** cortex/injection site. **(G)** Average cell body area of microglia in the perirhinal cortex, **(H)** CA1, and **(I)** cortex/injection site. **(D–I)** Data are presented as mean ± SEM. Data were analyzed using a two-way ANOVA (see Table 3). Statistically significant *post hoc* comparisons are indicated by bars with corresponding *p*-values. WT *n* = 4; TNF-α KO *n* = 5.

The perimeter and area of randomly selected microglia were measured (Figure 3A). In individual microglia isolated from 40× images, dividing microglia had longer cell body perimeters than non-dividing microglia in both WT and TNF-α KO mice (Figure 3B; see Table 2 for corresponding coefficient estimates and *p*-values). Microglial cell body area was also larger in dividing microglia compared to non-dividing microglia in WT and TNF-α KO mice (Figure 3C). No differences in the average cell body perimeter of microglia between hemisphere or genotype were found in the perirhinal cortex (Figure 3D), CA1 (Figure 3E), or cortex/injection site (Figure 3F; see Table 3 for corresponding *p*-values and F-statistics). There were no differences in the average microglia cell body area between genotype or hemisphere in the perirhinal (Figure 3G) or CA1 (Figure 3H) regions. However, cell body areas were larger in the cortex/injection site of the ipsilateral cortex compared to the contralateral cortex in both WT and TNF-α KO (Figure 3I).

**TABLE 2:** Coefficient estimates from generalized linear mixed models that tested differences in cell perimeters and areas between genotype × KI67 combinations.

Comparison	Perimeter		Area	
	Estimate	*P*-value	Estimate	*P*-value
KO KI67– vs. KO KI67+	–0.22	**< 0.01**	–0.51	**< 0.01**
KOKI67– vs. WT KI67–	–0.19	0.21	–0.16	0.34
KO KI67+ vs. WT KI67+	–0.13	0.66	–0.06	0.94
WT KI67– vs. WT KI67+	–0.16	**< 0.01**	–0.41	**< 0.01**

Statistically significant results are in bold text.

**TABLE 3:** ANOVA results from perimeter and area data (Figure 3) separated by brain region, histological stain, and effect.

Effect	Perirhinal		CA1		Cortex/injection site	
	Perimeter	Area	Perimeter	Area	Perimeter	Area
Hemisphere	*F*_1,14_ < 0.01 *p* = 0.95	*F*_1,14_ = 0.05 *p* = 0.83	*F*_1,14_ = 0.65 *p* = 0.43	*F*_1,14_ = 3.13 *p* = 0.10	*F*_1,14_ = 1.80 *p* = 0.20	*F*_1,14_ = 31.18 ***p* < 0.01**
Genotype	*F*_1,14_ = 0.37 *p* = 0.56	*F*_1,14_ = 1.04 *p* = 0.32	*F*_1,14_ = 0.72 *p* = 0.41	*F*_1,14_ = 0.50 *p* = 0.49	*F*_1,14_ < 0.01 *p* = 0.98	*F*_1,14_ = 0.42 *p* = 0.53
Interaction	*F*_1,14_ = 0.70 *p* = 0.42	*F*_1,14_ = 0.13 *p* = 0.72	*F*_1,14_ < 0.01 *p* = 0.96	*F*_1,14_ = 0.37 *p* = 0.55	*F*_1,14_ = 0.79 *p* = 0.39	*F*_1,14_ = 0.40 *p* = 0.54

Statistically significant results are in bold text.

### The morphology of dividing microglia was less complex

**FIGURE 4:**
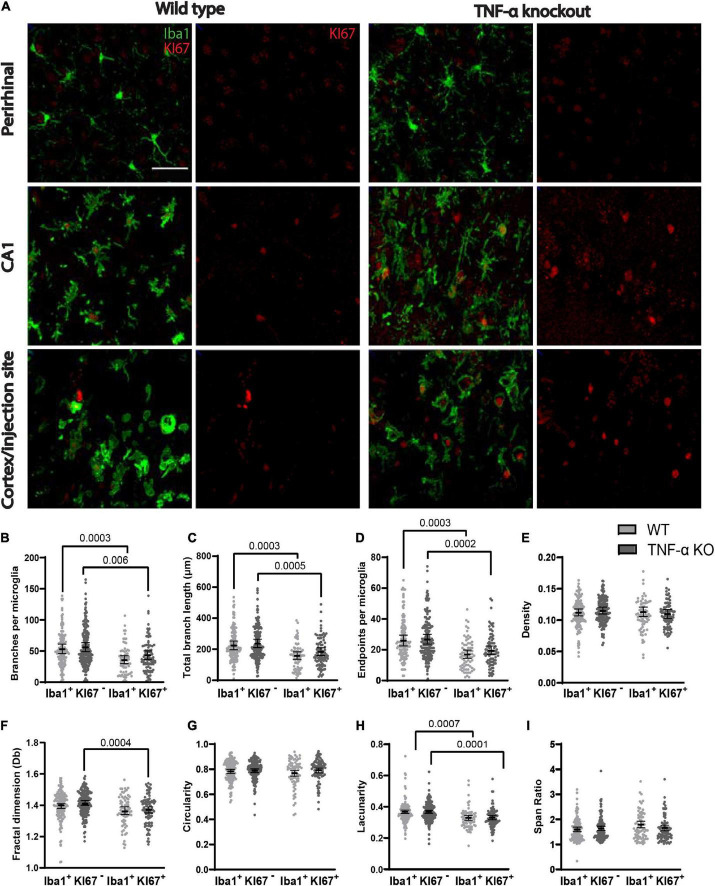
Dividing (KI67^+^) microglia had less complex, activated morphologies compared to microglia that were not dividing (KI67^–^). **(A)** Z-stacked photomicrographs of Iba1 (green) stained microglia and KI67 (red) stained dividing cells in the ipsilateral perirhinal cortex, CA1, and cortex/injection site in WT and TNF-α KO mice. **(B)** Total number of branches per microglia. **(C)** Total branch length per microglia. **(D)** Total number of endpoints per microglia. **(E)** Density of pixels per microglia. **(F)** Fractal dimension (Db) of microglia. **(G)** Circularity of microglia. **(H)** Lacunarity of microglia. **(I)** Span ratio of microglia. Individual data points are presented as dots and were analyzed using linear mixed models (see Tables 4, 5). Results are presented as point estimates with 95% confidence intervals. Statistically significant *post hoc* comparisons are indicated by bars with corresponding *p*-values. Iba1^+^/KI67^+^ = colocalization of Iba1 and KI67. TNF-α KO KI67^–^ *n* = 181 microglia; WT KI67^–^ *n* = 139 microglia; TNF-α KO KI67^+^ *n* = 87 microglia; WT KI67^+^ *n* = 71 microglia. Scale bar = 50 μm.

We compared the branching pattern and the morphological complexity of dividing and non-dividing microglia (Figure 4A). Dividing microglia had fewer branches per microglia (Figure 4B), a shorter total branch length (Figure 4C), and fewer branch endpoints per microglia (Figure 4D) compared to microglia that were not dividing (see Table 4 for corresponding coefficient estimates and *p*-values). There were no differences in the density of pixels per microglial cell between dividing microglia and microglia that were not dividing (Figure 4E; see Table 5 for coefficient estimates and *p*-values). Dividing microglia had a lower fractal dimension than microglia that were not dividing (Figure 4F). There were no differences in the circularity of microglia (Figure 4G). In both WT and TNF-α KO mice, dividing microglia had a lower lacunarity than microglia that were not dividing (Figure 4H). There were no differences in span ratio (cell shape/elongation) of microglia (Figure 4I). Although we found strong support that most dividing microglia had a more reactive morphology than microglia that were not dividing, the dispersion of the data points shown in Figure 4 also indicates that not all dividing microglia had a reactive morphology some microglia had a more ramified morphology than others during division.

**TABLE 4:** Coefficient estimates from generalized linear mixed models that tested differences in skeletal analysis metrics between genotype × KI67 combinations.

Comparison	Number of branches		Total branch length		Number of endpoints	
	Estimate	*P*-value	Estimate	*P*-value	Estimate	*P*-value
KO KI67– vs. KO KI67+	0.26	**< 0.01**	0.26	**< 0.01**	0.32	**< 0.01**
KOKI67– vs. WT KI67–	0.05	0.95	0.06	0.91	0.03	0.98
KO KI67 + vs. WT KI67 +	0.19	0.39	0.15	0.50	0.14	0.59
WT KI67– vs. WT KI67 +	0.40	**< 0.01**	0.35	**< 0.01**	0.42	**< 0.01**

Statistically significant results are in bold text.

**TABLE 5:** Coefficient estimates from generalized linear mixed models that tested differences in fractal analysis metrics between genotype × KI67 combinations.

Comparison	Density		Fractal dimension		Circularity		Lacunarity		Span ratio	
	Estimate	*P*-value	Estimate	*P*-value	Estimate	*P*-value	Estimate	*P*-value	Estimate	*P*-value
KO KI67– vs. KO KI67+	0.06	0.49	0.05	**< 0.01**	–0.02	0.99	0.16	**< 0.01**	–0.01	0.99
KO KI67– vs. WT KI67–	0.03	0.86	0.02	0.48	0.03	0.97	–0.01	0.99	0.02	0.94
KO KI67+ vs. WT KI67+	–0.03	0.92	0.004	0.99	0.13	0.40	0.01	0.99	–0.08	0.42
WT KI67– vs. WT KI67+	–0.01	0.99	0.03	**0.05**	0.09	0.62	0.17	**< 0.01**	–0.11	**0.03**

Statistically significant results are in bold text.

### Microglial morphology was most reactive near the injection site

**FIGURE 5:**
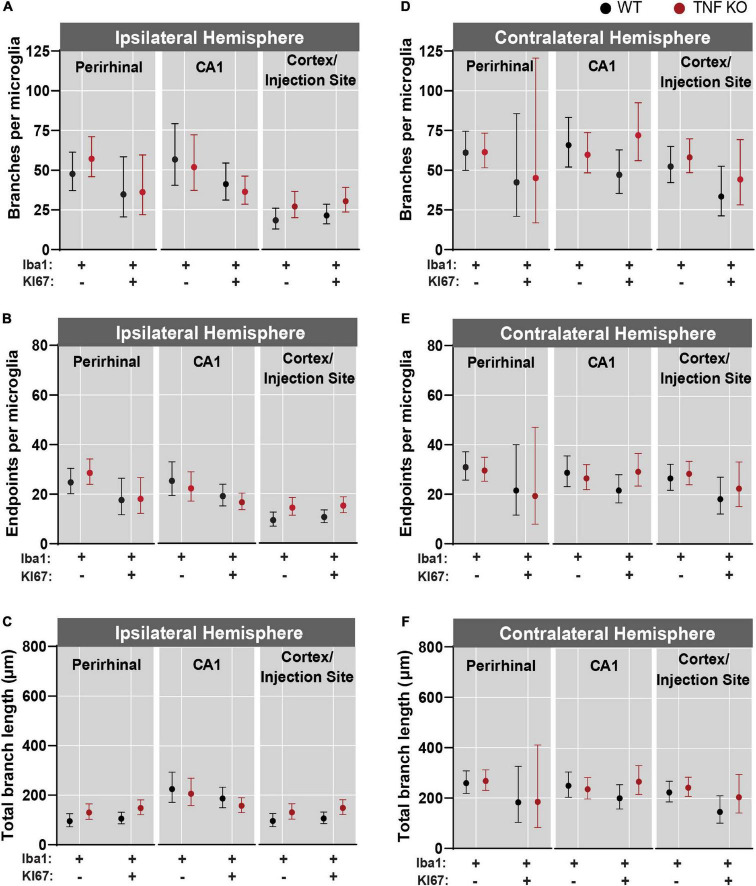
Microglial activation was greatest near the kainic acid injection site. Microglia were isolated from the perirhinal cortex, CA1, and cortex/injection site and data were analyzed across regions from both the ipsilateral and contralateral hemispheres. **(A)** Branches, **(B)** endpoints, and **(C)** total branch length per individual microglia isolated from the ipsilateral hemisphere. **(D)** Branches, **(E)** endpoints, and **(F)** total branch length per individual microglia isolated from the contralateral hemisphere. All data were analyzed using linear mixed models and results are presented as point estimates with 95% confidence intervals (see Supplementary Tables 1–3). TNF-α KO KI67^–^ *n* = 181 microglia; WT KI67^–^ *n* = 139 microglia; TNF-α KO KI67^+^ *n* = 87 microglia; WT KI67^+^ *n* = 71 microglia.

There was more microglial reactivity in the ipsilateral hemisphere (Figures 5A–C) than in the contralateral hemisphere (Figures 5D–F and see Supplementary Tables 1–3 for corresponding coefficient estimates and *p*-values). Within the ipsilateral hemisphere, microglia had the fewest number of branches and endpoints in the cortex (Figures 5A,B). Microglia in the ipsilateral CA1 had a longer total branch length than in the cortex and perirhinal regions (Figure 5C). In the contralateral hemisphere, there were no differences in branches per microglia, endpoints per microglia or total branch length per microglia across regions (Figures 5D–F).

## Discussion

**FIGURE 6:**
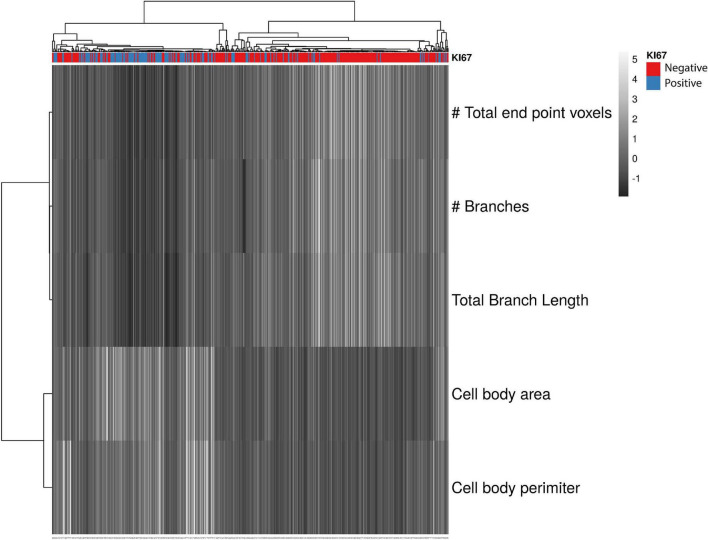
Summary Figure showing that KI67^+^ microglia have a more reactive morphology than KI67– microglia. Heatmap showing branch and cell body data clustered using correlation distance and average linkage. Unit variance scaling was applied to all continuous variables. Columns represent individual microglial cells. A score of –1 (blue) denotes a lower number and a score of 5 a higher number in the respective row. The majority KI67^–^ microglia had higher scores for skeletal analysis measurements (endpoints/microglia, branches/microglia and total branch length/microglia) and lower scores for cell body area and perimeter. Most KI67^+^ cells (red) had lower scores for skeletal analysis measurements and higher scores for cell body area and perimeter, indicating that dividing microglia had less ramification and a larger cell body. TNF-α KO KI67^–^ *n* = 181 microglia; WT KI67^–^ *n* = 139 microglia; TNF-α KO KI67^+^ *n* = 87 microglia; WT KI67^+^ *n* = 71 microglia.

It is critical that microglial cell division (i.e., mitosis) and reactivity are understood. Microglia can become reactive and release pro-inflammatory mediators after an insult or injury, which worsens functional outcomes. The reactivity and cell division of microglia rely on molecular pathways that include TNF-α (Kuno et al., 2005; Kong et al., 2019; Jang et al., 2021). Therefore, we investigated the morphology of dividing microglia and the effect of TNF-α on these morphologies. To date, a detailed, quantitative account of dividing microglial morphology has not been published. We found that most dividing microglia had a less complex morphology following administration of kainic acid. Classically, this less complex morphology has been associated with microglial reactivity in response to inflammatory stimuli (Badoer, 2010; Loane and Kumar, 2016; Morrison et al., 2017; Green et al., 2021). These data support published studies that found microglia divide in a reactive/amoeboid morphology (Alliot et al., 1999; Zusso et al., 2012). We also observed dividing microglia with complex, more ramified morphologies. We speculate that dividing microglial morphologies occur on a continuum, which may explain the range in morphologies we observed in dividing microglia (Figure 6).

Interestingly, in the contralateral perirhinal cortex, the region furthest away from the injection site, we found a greater range in skeletal analysis data from KI67^+^ microglia, compared to KI67^+^ microglia from the CA1 and cortex near the injection site. This large range in the number and length of microglial branches, and number of endpoints per microglia, indicates that dividing microglia had varying morphological complexities. We conclude that remote to the injection site, some dividing microglia had a reactive morphology, while other dividing microglia had a more ramified morphology. We must also consider that microglial division may be accompanied by morphological changes, regardless of their reactivity (Askew et al., 2017). These results are in agreement with two divergent hypotheses, that microglia can undergo cell division in both a ramified morphology (Glenn et al., 1992; Kloss et al., 1997) and a reactive/ameboid morphology (Alliot et al., 1999; Zusso et al., 2012). Under non-inflammatory conditions, microglia have a constant turnover rate (Réu et al., 2017). The population of microglia in the brain is dynamic and continuous turnover occurs to maintain the microglial population (Wirenfeldt et al., 2007; Askew et al., 2017). This maintenance involves a fine balance of cell division and apoptosis (Wirenfeldt et al., 2007; Askew et al., 2017). After an inflammatory insult, this balance is offset, and the number of microglia rapidly increases (Streit, 2006). It is possible that the ramified dividing microglia, found predominantly in cortices remote to the injection site, are dividing as part of this normal microglial turnover. However, under non-inflammatory conditions, the proportion of dividing microglia that have a ramified morphology versus ameboid morphology is unknown and this research question is an important future direction. An enduring debate among researchers is whether microglia can self-renew *via* cell division, or their population is replenished by infiltrating immune cells from the periphery that differentiate into microglia (Ginhoux et al., 2013). In agreement with previous research, our data suggest that microglia can divide to maintain their population (Ajami et al., 2007; Askew et al., 2017; Réu et al., 2017; Tay et al., 2017; Huang et al., 2018). However, we did not directly test if there was any contribution from infiltrating peripheral cells, CNS progenitor cells (Elmore et al., 2014), or perivascular macrophages (Masuda et al., 2022), which warrants additional research on microglia division.

Our findings support that kainic acid is a potent inflammatory trigger that results in microglia reactivity and division (Chang et al., 2006; Christensen et al., 2006; Akahoshi et al., 2007; Avignone et al., 2008; Ulmann et al., 2013; Bosco et al., 2018; Morin-Brureau et al., 2018; Feng et al., 2019; Araki et al., 2020; Di Nunzio et al., 2021). Not only can kainic acid injection induce seizures, which are associated with microglial reactivity, *in vitro* studies have shown that microglia can express kainic acid receptors (Noda et al., 2000). Therefore, there are multiple mechanisms through which kainic acid administration can cause microglial reactivity. As expected, we found the greatest number of dividing microglia in the cortex near the injection site, which may reflect a multimodal stimulation of microglial reactivity: the inflammation caused by the needle insertion, microglial reactivity following seizures, and reactivity induced directly by the kainic acid through microglia-specific receptors. Also, microglia near the injection site had larger cell bodies and a less ramified (i.e., reactive) morphology. In other studies that use an intraperitoneal injection of kainic acid, microglia were activated in the hippocampus when measured using microglia cell counts and percentage coverage of microglia (Avignone et al., 2008; Fox et al., 2020). A higher number of microglial cells corresponds to a higher percentage coverage of Iba1 + staining, which is interpreted as greater microglial reactivity. Our findings, using the intracerebral injection of kainic acid, did not produce the same conclusion as these intraperitoneal studies. This discrepancy in results may be because we did not consider the total expression of the marker Iba1, but instead used detailed quantitative analyses of morphology. This demonstrates how different methods can affect the outcomes of a study. Theoretically, microglia in the hippocampus were exposed to epileptiform activity caused by the kainic acid injection, which we hypothesize may have resulted in a different temporal and morphological response compared to microglia in the cortex near the injection site. Microglia near the injection site were exposed to both kainic acid and tissue injury caused by the insertion of the needle. We expect that the needle insertion disrupted tissue and increased reactivity and proliferation of local microglia. We postulate that discrepancies in the pathological stimuli between the CA1, and the cortex near the injection site, may account for the differences observed in microglial morphologies. We found a greater number of cells in the ipsilateral cortex, compared to the contralateral cortex, but we did not find a difference in the number of microglia in the hippocampus between hemispheres at 3 days post-injection. However, the microglia could have been reactive across both hemispheres. We postulate that both hemispheres of the hippocampus had similar levels of microglial reactivity because seizures propagate from the ipsilateral hippocampus, the epicenter of epileptiform activity after kainic acid injection, to the contralateral hemisphere of the hippocampus (Pernot et al., 2011; Bedner et al., 2015). The microglial reactivity induced by epileptic activity in the hippocampus could also explain discrepancies in the morphology of hippocampal microglial compared to cortical microglia. It is possible that the frequency and severity of epileptiform activity could contribute to the reactivity and proliferation of microglia. However, we had low variability in the reactivity of microglia in the WT KI67^+^ group, which suggests either that there was a low variability in the severity of seizures or that microglia morphological reactivity may not be dependent on the severity of epileptiform activity. Nevertheless, future studies using electroencephalogram recordings or seizure scoring are warranted to investigate the relationship between epileptiform activity and microglial reactivity (Feng et al., 2019; Wu W. et al., 2020).

Kainic acid administration increases microglial reactivity and proliferation (Ulmann et al., 2013; Feng et al., 2019). Microglial reactivity has been shown by elevated levels of CD11b staining, in the cortex and hippocampus but not cerebellum (Bockstael et al., 2014). Measuring the gross expression level of CD11b does not allow for morphometric analyses on Iba1 stained microglia, but our results are complimentary to this study as both show greater reactivity of microglia within close proximity to the injection site. Our results are also in agreement with studies that report microglial reactivity after kainic acid administration using a visual scoring system of microglial reactivity (Chen et al., 2005) and quantification of percentage area of microglia (Abraham et al., 2012).

A lack of sensitive and reliable methods to screen microglial morphology has prevented full comprehension of how microglial morphology differs spatially throughout the brain in response to an insult or injury. Technological advances, such as single cell RNA sequencing, have provided a better understanding of microglial gene expression after different inflammatory triggers (Masuda et al., 2020), but detailed histological analyses are less common. Detailed histological data can be collected using visual scoring systems in which a researcher provides a score based on observed microglial morphology (Woollacott et al., 2020). Although the data generated from that approach are categorical or qualitative, they still provide insight about the reactivity status of microglia (Woollacott et al., 2020). Furthermore, many studies use a percentage coverage of microglia to quantify microglial activation (Abraham et al., 2012; Norden et al., 2016; Feng et al., 2020; Fox et al., 2020; Swanson et al., 2020). This method can show useful information about changes in microglia activation associated markers (e.g., Iba1, CD11b), but do not provide morphometric data. As an alternative approach, we produced a heatmap that conveys the quantitative differences between KI67^+^ and KI67^–^ microglia. We observed the greatest microglial reactivity in the cortex near the injection site of the ipsilateral cortex. We found lower reactivity in the ipsilateral perirhinal cortex, remote to the injection site, and the lowest reactivity in the contralateral perirhinal cortex. These expected results help validate skeletal and fractal analysis methods for identifying microglial reactivity through morphometric data collection and analyses. Our data indicate that fractal and skeletal analysis techniques, applied to single microglia isolated from photomicrographs, are effective in differentiating between microglial morphologies (summarized in Figure 6).

TNF-α levels sharply increase in the cortex and hippocampus in the hours following a kainic acid injection (Minami et al., 1991; Wu D. et al., 2020). Further, the level of TNF-α has been shown to be higher in the ipsilateral hippocampus, than in the contralateral hippocampus 24 h after unilateral intracortical kainic acid injection (Nikolic et al., 2018). Therefore, we investigated whether knocking out microglial release of TNF-α affected microglial reactivity. We found few effects on microglial division and activation in TNF-α KO mice compared to WT mice. This suggests that the TNF-α, released from microglia, does not play a critical role in microglial cell division after an inflammatory stimulus using kainic acid. As many cytokines are released after a kainic acid injection (Minami et al., 1991; Oprica et al., 2006; Caracciolo et al., 2011), the effect of TNF-α on cell division may be dampened by high levels of synergistic pro-inflammatory cytokines. Previous studies reported that pharmacologically targeting TNF-α release from microglia using quercetin inhibited microglial cell reactivity both *in vivo* and *in vitro* (Wu D. et al., 2020). Potential discrepancies between those findings and ours could be that different techniques were used to measure microglial reactivity, as well as potential off-target neuroprotective effects of quercetin. It is possible that knocking out TNF-α receptor (TNFR)1 and/or TNFR2 on microglia may yield a more robust response than knocking out TNF-α. The KO model utilized in our study allowed for TNF-α from other sources to bind the receptors on the microglial cell surface. However, the KO of TNFR1 on microglia enhanced microglial reactivity after administration of kainic acid (Lu et al., 2008). Together, these data suggest that TNF-α is involved with many cellular outcomes, including inflammation, tissue degeneration, cell survival, apoptosis, and necroptosis, and completely eliminating TNF-α signaling may be pathological (Jang et al., 2021).

Histological studies come with the limitation that some cells may be sliced in different planes so that the entire cell body is not captured in the image. To combat this, we took Z-stacked images that spanned the 40 μm tissue. We cut the tissue at 40 μm because microglia typically have a 30 μm width/diameter (Kettenmann and Verkhratsky, 2011), with the exception of rod microglia that have a longer cell body (Taylor et al., 2014). We only collected tissue at 3 days post-kainic acid injection to ascertain whether the morphology of dividing microglia changes temporally after a kainic acid injection. A limitation of our study is the lack of a saline control; however, we wanted to quantify and measure the morphology of reactive and dividing microglia triggered by an inflammatory stimulus, as seen in many clinical conditions. We used the perirhinal cortex, remote to the injection site, in the contralateral hemisphere to obtain less reactive microglia that could be compared to microglia near the injection site, where we expected a higher reactivity of microglia. Using three regions of interest with different proximities to the injection site allowed for the isolation of microglia with an extensive range of morphologies and ramification. However, when considering the regions used in this study, it should be noted that microglia have been shown to vary in density and morphology throughout different regions of the brain. Such morphological variability should be considered as a limitation of our self-control study design and future studies are warranted to investigate further comparisons made to a saline control.

In conclusion, skeletal and fractal analysis methods are valid for identifying microglial reactivity through morphometric data collection. We collected data using sensitive and quantitative microglia morphology analyses and found support for our hypothesis. Dividing microglia had an activated, less ramified morphology, compared to non-dividing microglia after kainic acid-induced inflammation. These findings contribute to the gap in knowledge about microglial morphologies and their population dynamics. Understanding microglial morphologies and division after an inflammatory stimulus are essential for the development of new therapeutics that target microglia in diseased states, such as acquired neurological injuries, neurodegenerative diseases, and aging.

## Data availability statement

The raw data supporting the conclusions of this article will be made available by the authors, without undue reservation.

## Ethics statement

All experiments were approved by the Ethics Committee of Languedoc Roussillon n°36 and the French Ministry of Research (APAFIS#9899-2017042514488653 v4).

## Author contributions

TG executed the experiments, led the data collection, constructed the figures, and led manuscript writing. SM analyzed data and reviewed and edited the manuscript. MM-M performed the surgeries, kainic acid injections, and reviewed and edited the manuscript. EA was responsible for conceptualization of the study design, execution of the experiments, and reviewing and editing the manuscript. RR assisted with data analysis, visualization, conceptualization of the study design, and writing and editing the manuscript. All authors contributed to the article and approved the submitted version.

## Abbreviations

ANOVA: analysis of variance
CI: confidence intervals
CNS: central nervous system
Db: box-dimension
Iba1: ionized binding adapter molecule 1
IP: intraperitoneal
KO: Knockout
NDS: Normal donkey serum
PBS: phosphate buffered saline
SEM: standard error of the mean
TNFR: Tumor necrosis factor alpha receptor
TNF-α: tumor necrosis factor alpha
WT: wildtype.
