# Supplementary material for: Reactive morphology of dividing microglia following kainic acid administration
Source: Front Neurosci. 2022 Sep 29;16:972138. doi: 10.3389/fnins.2022.972138 (PMC9556904; doi:10.3389/fnins.2022.972138)
Supplement: Supplementary file 1 [file Data_Sheet_1.PDF]

Supplementary Material

1 Supplementary Data

Outliers excluded: Fractal dimension (WT $KI67^-$ n=1, TNF-α KO $KI67^-$ n=1); Lacunarity (WT $KI67^-$ n=1, TNF-α KO $KI67^-$ n=1, WT $KI67^+$ n=1, TNF-α KO $KI67^+$ n=1); Density [TNF-α KO $KI67^-$ n=1, WT $KI67^+$ n=1); Span ratio (WT $KI67^-$ n=1, TNF-α KO $KI67^-$ n=1, WT $KI67^+$ n=1, TNF-α KO $KI67^+$ n=1); Circularity (WT $KI67^-$ n=1, WT $KI67^+$ n=1, TNF-α KO $KI67^+$ n=1); Number of branches (WT $KI67^+$ n=1, TNF-α $KI67^+$ n=1); Cell body area (TNF-α KO $KI67^-$ n=1, WT $KI67^+$ n=1); Perimeter (TNF-α KO $KI67^-$ n=1, WT $KI67^-$ n=1, TNF-α KO $KI67^+$ n=1); Microglia cell number (ipsilateral WT $KI67^-$ n=1).

2 Supplementary Figures and Tables

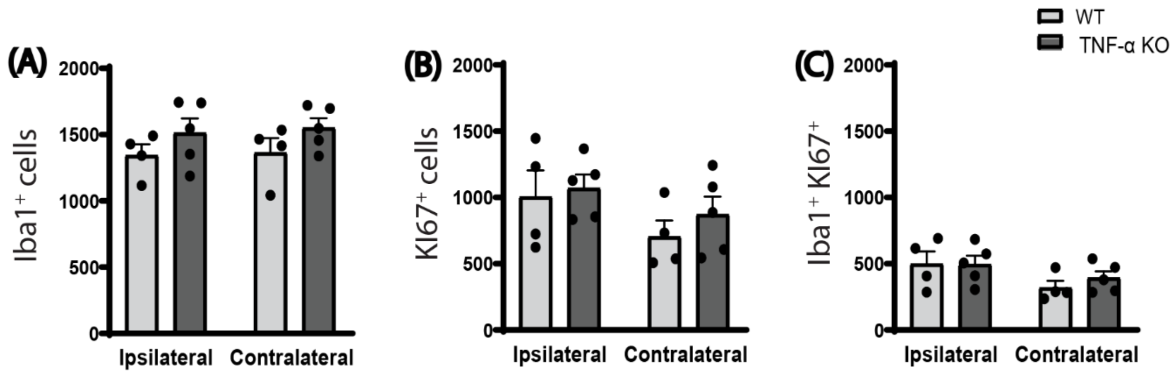

Supplementary Figure 1: There were no differences between genotypes or hemispheres in **(a)** the total number of microglia ($Iba1^+$ cells), **(b)**, total dividing cells ($KI67^+$), **(c)** or total number of dividing microglia ($Iba1^+$ & $KI67^+$) in our representative hippocampal slices.

Supplementary Table 1. Coefficient estimates from generalized linear mixed models that tested differences in number of branches per microglia among genotype×KI67×region combinations in each hemisphere.

	Ipsilateral		Contralateral	
**Comparison**	**Estimate**	***P*-value**	**Estimate**	***P*-value**
WT KI67- Peri vs. WT KI67+ Peri	0.32	0.95	0.37	0.93
KO KI67- Peri vs. KO KI67+ Peri	0.46	0.80	0.31	0.99
WT KI67- Peri vs. KO KI67- Peri	-0.18	0.96	-0.01	0.99
WT KI67+ Peri vs. KO KI67+ Peri	-0.04	0.99	-0.06	0.99
WT KI67- CA1 vs. WT KI67+ CA1	0.32	0.87	0.33	0.70
KO KI67- CA1 vs. KO KI67+ CA1	0.35	0.76	-0.19	0.88
WT KI67- CA1 vs. KO KI67- CA1	0.09	0.99	0.097	0.99
WT KI67+ CA1 vs. KO KI67+CA1	0.13	0.99	-0.42	0.58
WT KI67- Cortex vs. WT KI67+ Cortex	0.16	0.99	0.45	0.73
KO KI67- Cortex vs. KO KI67+ Cortex	-0.11	0.99	0.28	0.90
WT KI67- Cortex vs. KO KI67- Cortex	-0.39	0.82	-0.11	0.98
WT KI67+ Cortex vs. KO KI67+ Cortex	-0.35	0.77	-0.28	0.96
WT KI67- Peri vs. WT KI67- CA1	0.17	0.99	0.07	0.99
WT KI67- Peri vs. WT KI67- Cortex	-0.96	**0.0002**	-0.16	0.88
WT KI67- CA1 vs. WT KI67- Cortex	1.13	**0.0002**	0.23	0.80
KO KI67- Peri vs. KO KI67- CA1	-0.10	0.99	-0.03	0.99
KO KI67- Peri vs. KO KI67- Cortex	-0.75	**0.002**	-0.06	0.99
KO KI67- CA1 vs. KO KI67- Cortex	0.65	0.12	0.03	0.99
WT KI67+ Peri vs. WT KI67+ CA1	0.17	0.99	0.11	0.99

Supplementary Table 1 continued				
WT KI67+ Peri vs. WT KI67+ Cortex	-0.48	0.82	-0.24	0.99
WT KI67+ CA1 vs. WT KI67+ Cortex	0.65	**0.02**	0.34	0.85
KO KI67+ Peri vs. KO KI67+ CA1	0.002	0.99	0.46	0.95
KO KI67+ Peri vs. KO KI67+ Cortex	-0.18	0.99	-0.03	0.99
KO KI67+ CA1 vs. KO KI67+ Cortex	0.18	0.94	0.49	0.69

Supplementary Table 2. Coefficient estimates from generalized linear mixed models that tested differences in number of endpoints per microglia among genotype×KI67×region combinations in each hemisphere.

Comparison	Ipsilateral		Contralateral	
	**Estimate**	***P*-value**	**Estimate**	***P*-value**
WT KI67- Peri vs. WT KI67+ Peri	0.34	0.85	0.36	0.88
KO KI67- Peri vs. KO KI67+ Peri	0.46	0.50	0.43	0.91
WT KI67- Peri vs. KO KI67- Peri	-0.14	0.94	0.04	0.99
WT KI67+ Peri vs. KO KI67+ Peri	-0.03	0.99	0.11	0.99
WT KI67- CA1 vs. WT KI67+ CA1	0.28	0.76	0.29	0.72
KO KI67- CA1 vs. KO KI67+ CA1	0.29	0.68	-0.10	0.97
WT KI67- CA1 vs. KO KI67- CA1	0.13	0.99	0.82	0.99
WT KI67+ CA1 vs. KO KI67+ CA1	0.14	0.98	-0.30	0.77
WT KI67- Cortex vs. WT KI67+ Cortex	-0.13	0.99	0.38	0.76
KO KI67- Cortex vs. KO KI67+ Cortex	-0.05	0.99	0.24	0.87
WT KI67- Cortex vs. KO KI67- Cortex	-0.44	0.52	-0.07	0.99
WT KI67+ Cortex vs. KO KI67+ Cortex	-0.36	0.52	-0.21	0.98
WT KI67- Peri vs. WT KI67- CA1	0.02	0.99	-0.07	0.99
WT KI67- Peri vs. WT KI67- Cortex	-0.98	**0.0003**	-0.15	0.85
WT KI67- CA1 vs. WT KI67- Cortex	1.003	**0.0002**	0.08	0.99
KO KI67- Peri vs. KO KI67- CA1	-0.25	0.77	-0.12	0.89
KO KI67- Peri vs. KO KI67- Cortex	-0.69	**0.0001**	-0.04	0.99
KO KI67- CA1 vs. KO KI67- Cortex	0.44	0.27	-0.07	0.99
WT KI67+ Peri vs. WT KI67+ CA1	0.08	0.99	0.009	0.99

Supplementary Table 2 continued				
WT KI67+ Peri vs. WT KI67+ Cortex	-0.52	0.45	-0.17	0.99
WT KI67+ CA1 vs. WT KI67+ Cortex	0.59	**0.004**	0.17	0.96
KO KI67+ Peri vs. KO KI67+ CA1	-0.09	0.99	0.42	0.93
KO KI67+ Peri vs. KO KI67+ Cortex	-0.18	0.99	0.15	0.90
KO KI67+ CA1 vs. KO KI67+ Cortex	0.09	0.99	0.27	0.87

Supplementary Table 3. Coefficient estimates from generalized linear mixed models that tested differences in total branch lengths per microglia among genotype×KI67×region combinations in each hemisphere.

**Comparison**	**Ipsilateral**		**Contralateral**	
	**Estimate**	***P*-value**	**Estimate**	***P*-value**
WT KI67- Peri vs. WT KI67+ Peri	0.42	0.75	0.35	0.88
KO KI67- Peri vs. KO KI67+ Peri	0.37	0.79	0.37	0.94
WT KI67- Peri vs. KO KI67- Peri	-0.14	0.98	-0.03	0.99
WT KI67+ Peri vs. KO KI67+ Peri	-0.19	0.99	-0.01	0.99
WT KI67- CA1 vs. WT KI67+ CA1	0.19	0.95	0.22	0.80
KO KI67- CA1 vs KO KI67+ CA1	0.27	0.80	-0.12	0.92
WT KI67- CA1 vs. KO KI67- CA1	0.09	0.99	0.55	0.99
WT KI67+ CA1 vs. KO KI67+ CA1	0.17	0.94	-0.29	0.75
WT KI67- Cortex vs. WT KI67+ Cortex	-0.10	0.99	0.43	0.57
KO KI67- Cortex vs. KO KI67+ Cortex	-0.13	0.10	0.17	0.95
WT KI67- Cortex vs. KO KI67- Cortex	-0.31	0.81	-0.08	0.99
WT KI67+ Cortex vs. KO KI67+ Cortex	-0.34	0.51	-0.34	0.85
WT KI67- Peri vs. WT KI67- CA1	0.03	0.99	-0.03	0.99
WT KI67- Peri vs. WT KI67- Cortex	-0.83	**0.0002**	-0.15	0.83
WT KI67- CA1 vs. WT KI67- Cortex	0.06	0.99	0.11	0.91
KO KI67- Peri vs. KO KI67- CA1	-0.20	0.91	-0.12	0.86
KO KI67- Peri vs. KO KI67- Cortex	-0.66	**0.0001**	-0.10	0.90
KO KI67- CA1 vs. KO KI67- Cortex	0.46	0.26	-0.03	0.99
WT KI67+ Peri vs. WT KI67+ CA1	0.26	0.95	0.09	0.99

Supplementary Table 3 continued

WT KI67+ Peri vs. WT KI67+ Cortex	-0.31	0.90	-0.23	0.99
WT KI67+ CA1 vs. WT KI67+ Cortex	0.57	**0.007**	0.32	0.81
KO KI67+ Peri vs. KO KI67+ CA1	-0.09	0.99	0.37	0.95
KO KI67+ Peri vs. KO KI67+ Cortex	-0.16	0.99	0.10	0.99
KO KI67+ CA1 vs. KO KI67+ Cortex	0.06	0.99	0.27	0.85
